# Interlaboratory Study on the Lithographically Produced Scanning Electron Microscope Magnification Standard Prototype

**DOI:** 10.6028/jres.098.033

**Published:** 1993

**Authors:** Michael T. Postek, Andras E. Vladar, Samuel N. Jones, William J. Keery

**Affiliations:** National Institute of Standards and Technology, Gaithersburg, MD 20899-0001

**Keywords:** calibration, linewidth, lithography, magnification, pitch, scanning electron microscope, SEM, standard

## Abstract

NIST is in the process of developing a new scanning electron microscope (SEM) magnification calibration reference standard useful at both high and low accelerating voltages. This standard will be useful for all applications to which the SEM is currently being used, but it has been specifically tailored to meet many of the particular needs of the semiconductor industry. A small number of test samples with the pattern were prepared on silicon substrates using electron beam lithography at the National Nanofabrication Facility at Cornell University. The structures were patterned in titanium/palladium with maximum nominal pitch structures of approximately 3000 μm scaling down to structures with minimum nominal pitch of 0.4 (μm. Eighteen of these samples were sent out to a total of 35 university, research, semiconductor and other industrial laboratories in an interlaboratory study. The purpose of the study was to test the SEM instrumentation and to review the suitability of the sample design. The laboratories were asked to take a series of micrographs at various magnifications and accelerating voltages designed to test several of the aspects of instrument performance related to general SEM operation and metrology. If the instrument in the laboratory was used for metrology, the laboratory was also asked to make specific measurements of the sample. In the first round of the study (representing 18 laboratories), data from 35 instruments from several manufacturers were obtained and the second round yielded information from 14 more instruments. The results of the analysis of the data obtained in this study are presented in this paper.

## 1. Introduction

**Fig. 1:**
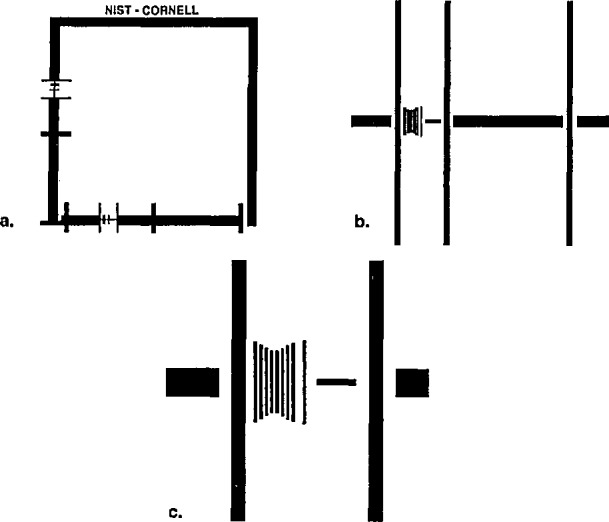
Drawings of the NIST prototype SEM pattern as written by the electron beam writing system for this study, (a) 1 mm pattern, (b) Medium magnification pitch pattern, (c) Highest magnification pitch pattern showing the 0.4 μm pitch. The large 3 mm pattern is not shown.

NIST is in the process of developing a new low accelerating voltage SEM magnification calibration reference standard [1]. This standard will be useful for all applications to which the SEM is currently being used, but it has been specifically tailored to meet many of the particular needs posed by the semiconductor industry. These needs have been outlined previously [2] but, specifically, include the need of the industry for sub-half micrometer calibration structures that are able to be used to calibrate the instrumentation at low accelerating voltages. The standard must be able to be inserted into and be used on the dedicated on-line wafer inspection instruments. The current NIST SEM magnification standard, Standard Reference Material (SRM) 484 was not designed for this purpose and does not meet all of these fundamental semiconductor industry needs. It should be noted, however, the new standard is not intended to replace SRM 484 but to supplement it where the need exists. The overall characteristics of the new prototype standard have been published previously [1,2] and since this description and proof of concept were published, work has been done to have this sample fabricated in bulk quantities. For this interlaboratory study, a number of test samples were contracted by NIST to be fabricated on silicon substrates using electron beam lithography at the National Nanofabrication Facility (NNF) at Cornell University. The prototype samples were patterned in titanium/palladium with maximum nominal pitch structures of approximately 3000 μm scaling down to structures with minimum nominal pitch of 0.4 μm (Fig. 1). It was necessary for the samples (for this study) to be fabricated in the titanium/palladium and at a larger minimum pitch geometry (0.4 μm) than the originally desired 0.2 (μm minimum pitch because of processing limitations at the NNF when this batch of samples was made. This compromise was not deemed a limitation to the interlaboratory study since the main purpose of the study was to have the pattern design reviewed in order to determine if any instrument specific modifications should be made to the pattern. Eighteen of the samples were sent out to a variety of university, research, semiconductor, and other industrial laboratories. This was done in two rounds since there were two sets of patterns available for testing on each sample. Thus, data were obtained from a total of 49 instruments.

This study is referred to as an interlaboratory study rather than a “round robin” because multiple test samples were used. The purpose of the study was to test the instrumentation and to determine the suitability of the sample design. The laboratories chosen were asked to submit to NIST a series of micrographs at specific magnifications and accelerating voltages designed to test several aspects of instrument performance related to SEM operation and metrology. If the instrument in the laboratory was used for metrology, the laboratory was also asked to make specific measurements of the sample.

## 2. Materials and Methods

### 2.1 Scanning Electron Microscopes

Imaging and measurements, for this work, were done by the participants on a variety of instrument types. The list of instrumentation is shown in Table 1, however; the performance of the instruments, as well as, the participants in the study will remain anonymous. This cross section of SEMs represented instruments as old as 15 years to modern instrumentation. Sample inspection and comparison work supporting this study at NIST was done with a Hitachi S-4000 field emission scanning electron microscope (FESEM).^1^ Measurements of the video signal were done on the FESEM using the beam scanned mode because the NIST metrology instrument [2] was unavailable during much of this study since being specially modified and equipped for x-ray mask measurements [3].

A limited amount of sample data was obtained from the NIST metrology instrument. The instrument was used in the stationary beam, sample scanned mode of operation described previously [2] with new software and hardware modifications [3]. For this work, the electron signal was collected using a solid state backscattered electron detector at high accelerating voltage (30 kV) and the measurement data were taken in the backscattered electron detection mode [4].

### 2.1.1 FESEM System

The prototype samples were sent out to the participants of the first round without initial SEM inspection in order to minimize any initial sample contamination. Upon their return, the samples were mounted on standard specimen stubs and carefully inserted into the Hitachi S-4000 instrument. Each sample was viewed at low accelerating voltage in order to assess the contamination level on the surface. The sample was then measured and photographed at high accelerating voltage. The image was also taken and stored in the “Isaac” System (described below) for image analysis. Any sample with excessive contamination was not sent out in the second round.

**Fig. 2:**
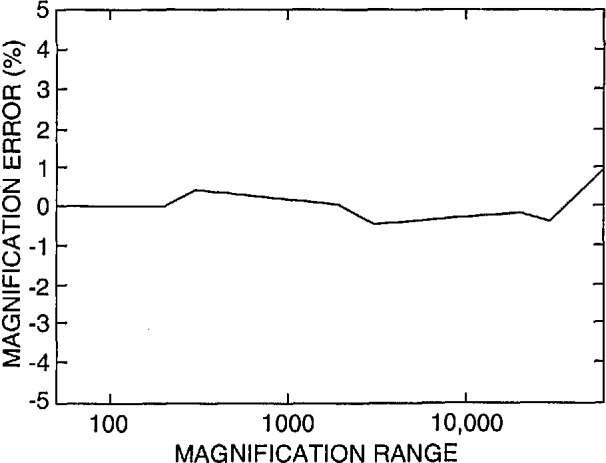
Plot of the magnification calibration error of the NIST SEM as related to the certified SRM 484 value.

The FESEM was accurately calibrated using NIST SRM 484 at high accelerating voltage (20keV) with a procedure developed at NIST using the Hitachi keyboard measurement system accessory. Adjustment of this instrument resulted in a calibration ±1% of the certified value for SRM 484 as shown in Fig. 2.

Unfortunately, with the instrument currently equipped, any imaging or measurement data were unable to be directly transferred to an ancillary computer system for image analysis. This transfer was necessary in order to analyze all the data (participants data and NIST data) in the same manner using the same algorithms. This necessitated the development of the system described below.

#### 2.1.2 “Isaac” Image Analysis System

A computer based measurement system christened “Isaac” was developed to analyze the SEM images from the Hitachi S-4000 FESEM, as well as other “scanned-in” or digitally obtained data.

##### Hardware

This system is based on an Apple Macintosh IIfx computer [5]. The images are captured with a high speed frame grabber, PIXEL PIPELINE card [6]. The video signal for the Isaac system is grabbed at TV frequency from the SEM (512×512) or scanned at 600 dots per 25.4 mm (600 dpi) into the computer using the scanner and then stored and manipulated in the computer system. The pixels of both the scanner and the Isaac have been calibrated with accurate NIST certified linear scales. A typical 512×512 SEM digital storage system functions at about 100 DPI. This means that in comparison, the scanned image is operating with about 5 times the pixel density. Barring any “blooming” of the photographic emulsion this provides a highly precise representation of the images submitted by the participants.

##### Software

The software generally used on the system is a commercially available scientific image analysis program called IP Lab Spectrum [7]. The IP Lab Spectrum program also has an extension developed by Signal Analytics in collaboration with NIST specially designed for linewidth or pitch measurements used in this work. The public domain program named “Image” of the National Institutes of Health [8] was also useful in this work. With Image and IP Lab Spectrum, there is the capability to control the frame grabber card, and then use the built-in tools, modifications, pseudo-colorisation, calculations, measurements and other features. For control of the image scanning the commercially available program “Adobe Photoshop” was used.

Further improvements of both the hardware and the software the Isaac system for higher resolution digitization are currently in progress.

### 2.2 Experiment

#### 2.2.1 Instrument Conditions

Scanning electron microscopes are operated in a variety of manners depending on the laboratory. Some are exclusively low voltage instruments such as many of those used in the semiconductor industry for on-line inspection while others are exclusively high voltage instruments. Many general laboratory instruments operate through both extremes depending on the work to be done. Because of the variety of participants chosen for this study several experimental possibilities were offered. The participants were asked either to do the high accelerating voltage set of micrographs, a low accelerating voltage set of micrographs or both sets of micrographs. The instrument was expected to be operating with conditions optimized for the chosen operation range. All of the micrographs and/or measurements were to be done at 0° tilt (normal incidence to the electron beam).

#### 2.2.2 Accelerating Voltage

All micrographs or measurements were to be made at nominal (what the instrument indicates) 1 and 5 kV for the low accelerating voltage set and nominal 10 and 30 kV for the high accelerating voltage set.

#### 2.2.3 Magnification Ranges

Example micrographs of the requested pattern sites at the magnifications requested were provided for each accelerating voltage set. The eight magnification ranges established are shown in Table 2. These ranges were chosen to demonstrate the decade magnification calibration of the instruments [2] and the two sets of accelerating voltage were chosen to demonstrate any magnification variation due to failure of the instrument compensation system to correct for changes in accelerating voltage. Lens hysteresis effects on the magnification would be minimized, in this particular study, *if* the participants followed the directions provided and worked from low accelerating voltage to high accelerating voltage and not the converse.

If the instrument was not able to operate at the higher accelerating voltages (5 kV and above), such as in the newer wafer inspection instruments, the participant was asked to do the 1 kV work and then use the highest accelerating voltage available (i.e., still provide two sets of data). Since performance between the various classes of instruments varied, it was fully understood and appreciated that some instruments were are not capable of doing all of the experimental magnifications requested (i.e., an instrument equipped with a tungsten filament would not be expected to provide a good Range 8 or 100 000 × micrograph at 1 kV). All the participants were requested to provide the best quality micrographs for the evaluation.

#### 2.2.4 Measurement System

If the instrument was equipped with a linewidth type measurement system the participants were asked to provide a hardcopy of the measurement data for each micrograph and wherever possible an ASCII dump of the data for NIST analysis on disk (IBM or Macintosh compatible).

### 2.3 Prototype Standard

#### 2.3.1 Magnification Standards

**Fig. 3:**
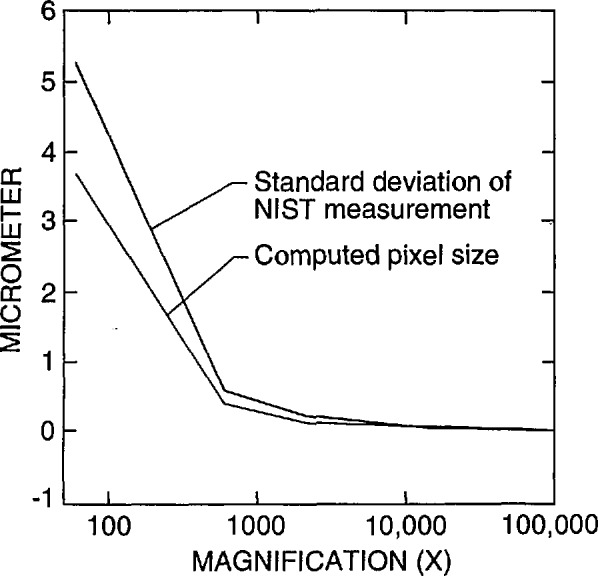
Plot of the standard deviation of the NIST instrument measurement of all the samples successfully returned to the computed pixel size relative to the magnification ranges surveyed.

Currently, the only certified magnification standard available for calibration of the magnification of an SEM is NIST SRM 484. SRM 484 is composed of thin gold lines separated by layers of nickel providing a series of pitch structures ranging from nominally 1 to 50 μm [9] (depending on the version). This standard is still very viable for many SEM applications. Certain limitations presented by this standard for the semiconductor industry have been published previously [2]. The prototype standard in this test was designed to minimize or eliminate the limitations of SRM 484 for calibration of instruments used in the semiconductor industry. Since this was an interlaboratory comparison study and not the issuance of a standard, the samples were carefully measured only in the FESEM using beam scanning mode and the images acquired into the Isaac system in slow scan mode. The FESEM (and the Isaac) was calibrated accurately in slow scan mode using the NIST SRM 484 at high accelerating voltage. This provided a computed calibration error for the SEM in the “X” direction of only about ± 1% as compared to the certified measurements on SRM 484 (Fig. 2). This error could be reduced by finer steps in the electronics of the magnification calibration system. All the comparison measurements of the participant’s samples were made at the same FESEM calibrated accelerating voltage and working distance. Measurement with the FESEM of the samples returned to NIST using this procedure resulted in a measurement precision with a standard deviation of no greater than about +1 pixel width over the entire measurement range (Fig. 3). Each new standard, when issued, will be individually calibrated using the NIST metrology SEM thus providing a certified, NIST traceable measurement of the spacing (or pitch) between the various lines making up the standard.

#### 2.3.2 Measurement Criteria

Most modern scanning electron microscopes provide an alphanumeric display of the magnification and a micrometer bar on the viewing screen. These data are also recorded on the micrograph. Measurement data are obtained directly from the image, the micrograph (as a unit) or from a digital measurement system. The confidence we can place on the accuracy of those readouts depends upon many factors—the main one being magnification (column scan) calibration. The semiconductor industry today, relies greatly upon the measurements made in scanning electron microscopes to control million dollar process lines. However, the correctness of the answer to the question of “How big is it?” relates directly to two major factors in the SEM, as well as a whole host of lesser factors [10]. The first and foremost factor is the accurate magnification calibration of the SEM. Magnification, in an SEM, is essentially defined as the ratio between the area scanned by the electron beam on the specimen to the area displayed or photographed or measured. It is imperative that the distance being scanned by the electron beam be accurately calibrated.

**Fig. 4:**
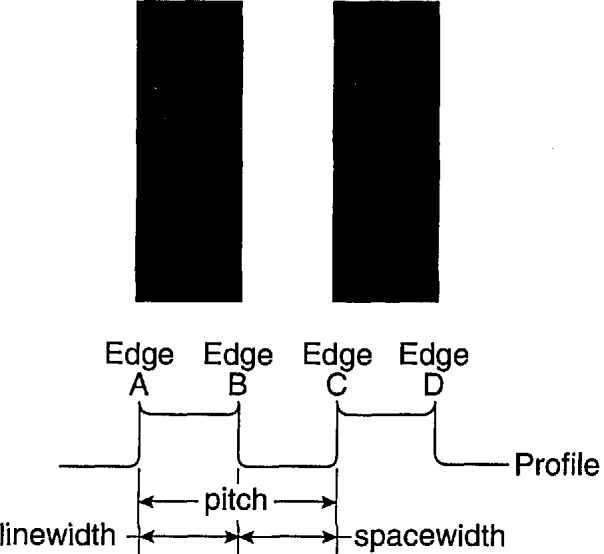
Graphic comparison between the measurement of pitch and width. Measurement of A to C or measurement of B to D defines the pitch of the sample. Measurement of A to B or C to D defines the linewidth of the sample and measurement of B to C defines the spacewidth.

The second factor relating to SEM measurements is the effect on the image induced by the electron beam/specimen interaction. This factor cannot be ignored. Fortunately it can be minimized by the use of a “pitch” type magnification calibration sample, such as SRM 484, or this new standard when it is issued. These standards are both based on the measurement of “pitch.” A pitch is the distance from the edge of one portion of the sample to a similar edge some distance away from that first edge (Fig. 4). In Fig. 4, a measurement of the pitch would be the distance from A to C or from B to D. In a pitch standard, that distance is certified and it is to that certified value that the magnification calibration of the SEM is set. If we consider two lines separated by some distance, the measurement of the distance from the leading edge of the first line to the leading edge of the second line defines the pitch. Many systematic errors included in the measurement of the pitch are equal on both of the leading edges; these errors, including the effect of the specimen beam interaction, cancel. This form of measurement is therefore self-compensating. The major criteria for this to be a successful measurement is that the two edges measured must be similar in all ways. SEM magnification can be easily and accurately calibrated to a pitch using SRM 484, the NIST certified magnification calibration standard or this standard when issued.

The measurement of a width of one of the lines, on the other hand, (A to B or C to D on Fig. 4), is complicated in that many of the errors (vibration, electron beam interaction effects, etc.) are now additive. Therefore, errors from both edges are included in the measurement. SEM magnification should not be calibrated to a width measurement since these errors vary from specimen to specimen due to the differing electron beam/sample interaction effects. Effectively, with this type of measurement we do not know the accurate location of an edge in the video image and more importantly it changes with instrument conditions (this can be seen later in Sec. 3.4). The determination of the edge location requires electron beam modeling of the interactions occurring both in the sample and the specimen chamber, as well as, modeling of the electron collection. This is the ultimate goal of this program and recently has been shown to be successful for special samples such as x-ray masks measured in the SEM [3].

#### 2.3.3 “X” and “Y” Magnification Calibration

The “X” and the “Y” scans of an SEM must be independently calibrated in order that round objects appear round and square objects appear square. That is to say, measurements of a defined pitch in the X direction must agree with measurements of the same structure (physically rotated by 90°) in the Y direction. For this study, all measurements were to be made in the “X” direction. The first group of participants were only concerned with the pattern located in the “X” direction. Therefore, no direct determination of the squareness of the X to Y calibration was done by the participants. However, these data could be obtained from the lowest magnification images supplied (see Sec. 3). The pattern in the “X” direction is defined as the one parallel to the NIST-CORNELL label (see Fig. 1). The second group using the same samples were asked to measure the features located in the “Y” direction which is perpendicular to the label (since these presumably had not been contaminated by previous scans), but measured in the “X” direction by inserting the pattern and physically rotating it into position.

#### 2.3.4 Sample Materials

**Fig. 5:**
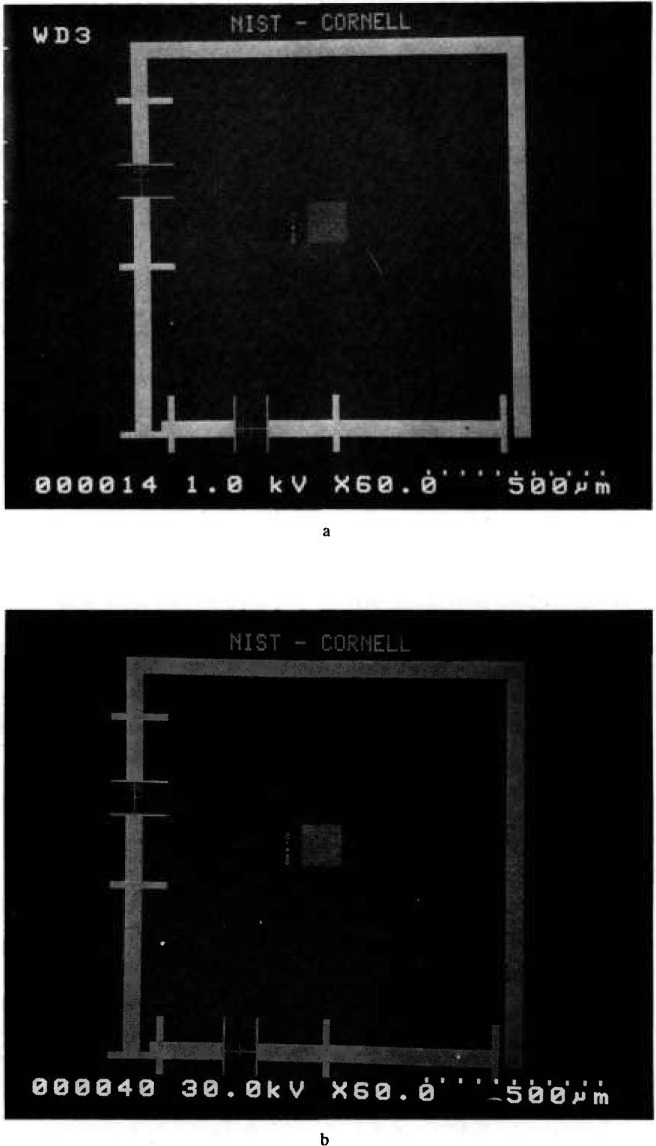
NIST prototype SEM magnification sample, (a) Low accelerating voltage image at 1 kV. (b) High accelerating voltage image at 30 kV.

The NIST sample was lithographically produced with an electron beam at the National Nanofabrication Facility at Cornell University. This sample was composed of titanium (10 nm) and palladium (50 nm) for a nominal thickness of about 60 nm on a standard silicon wafer. Future samples will be fabricated of the preferred heavy metal silicide. The sample works well at both high and low accelerating voltages (Fig. 5).

#### 2.3.5 Pattern

The prototype sample is composed of a large, approximately 3 mm (nominal) outer pattern and a smaller 1 mm inner pattern. Embedded in the smaller pattern is an array of calibration lines (Fig. 1) reducing in pitch, in steps, to a nominal 0.2 μm pitch. The large pattern is used to calibrate the SEM in the lower decades of the magnification range; whereas, the smaller patterns (as shown in Fig. 1) are used for the upper decades. Various combinations of these patterns might be used in a typical instrument calibration (Table 2). For a full instrument calibration of most instruments, several measured pitches of various structures would be used from the calibration sample. For the full range of magnifications to be properly calibrated, several steps progressing from low magnification to high magnification may require adjustment first — then the offset calibrated at a high magnification step. This procedure will vary with the instrument design. The current prototype sample has calibration patterns written in both the “X” and the “Y” directions in order to permit the full calibration of the X and the Y scans of the SEM without physical sample rotation. Raster rotation is not a proper procedure for use during magnification calibration because this circuitry can, in some instances, distort the X and the Y scans.

The NIST prototype sample was designed for use in the standard “post-lens type” SEM where the sample is found below the lens. This is typical of most laboratory and many production instruments. Special “in-lens type” SEMs where the sample is inserted into the final lens, generally require smaller samples since the available space within the lens is quite restricted. The prototype sample was viewed in one in-lens type instrument, but, the placement of the sample within the instrument required breaking the sample into a smaller piece. For the in-lens type instruments, future versions of this magnification sample could be made having only the inside 1 mm calibration pattern. This would significantly reduce the size and would not compromise the calibration function since low magnification operation (where calibration using the larger 3 mm pattern is needed) in these microscopes is not possible.

**Fig. 6:**
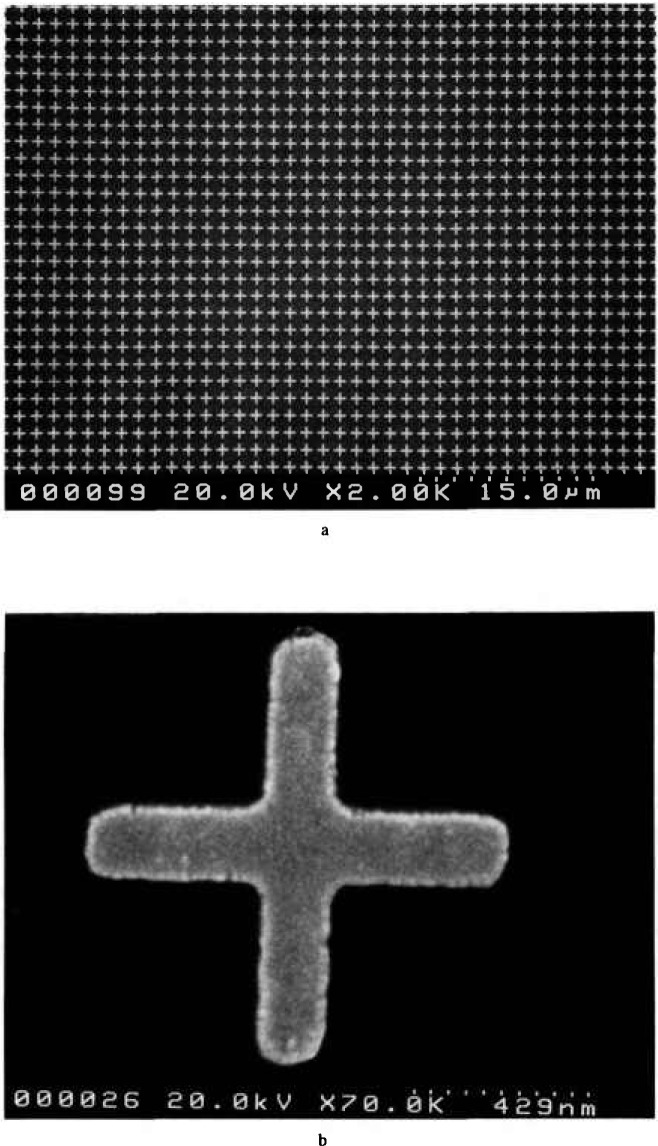
Focus and astigmatism correction structures located in the center of the 1 mm pattern (a) Low magnification, (b) High magnification.

Included in the center of the 1 mm pattern, is a matrix of small crosses used to focus and correct the astigmatism of the electron beam. These structures are used for instrument set-up; then the field is moved over to the actual pattern for the final measurement work (Fig. 6).

#### 2.3.6 Sample Mounting

The NIST sample was pre-diced from the wafer into approximately 12 mm squares each holding a single complete pattern. For standard inspection or research-type SEMs, the sample was mounted, with carbon-based adhesive, on any platform or stub required by the particular instrument.

Mounting of the sample for the new dedicated wafer inspection instruments presented a slightly more difficult problem. Placement of the sample on the surface of a wafer the proper size for the instrument was acceptable if the added thickness of the NIST sample did not compromise the working distance/magnification compensation system of the instrument. This means that if the instrument expects the wafer to always be at a certain working distance for focus (and therefore magnification compensation and computation) it may not be able to accommodate the difference in the magnification resulting from the added thickness of the specimen/wafer. If there was any question, the participants were asked to contact the SEM manufacturer. Alternatively, a specially prepared sample was inserted into a conductive 150 mm (6 in) wafer, flush with the surface. This sample holder was made available to the participants upon request.

#### 2.3.7 Specimen Contamination

It was inevitable that the samples would become contaminated from handling and from the vacuum system of the instrument. Sample contamination is especially troublesome at low accelerating voltages. Therefore, those participating in the low accelerating voltage aspects of the study were asked to make the low accelerating voltage micrographs first (starting at low magnification) and then work up in the proper steps to the high accelerating voltages and magnifications. In order to minimize contamination during the inspection phase, the NIST FESEM was equipped with a special liquid nitrogen cold trap and a nitrogen leak system.

## 3. Results

The participants of the study provided NIST with micrographs and data in several formats. In some instances the data were supplied in as varied media as “instant” film, video prints and optical disks. Except for the digital storage (which may have its own artifacts in the form of digitization noise), it is fully understood that the recording of the data in these formats can introduce artifacts. For example it is reasonably well known that “instant” film can shrink and change dimensions during the development process. However, it was necessary to work with the data and media provided. This is also sensible since, in common operation, many important conclusions are based on the same type of data format.

Two major studies were done on the data submitted. The first was an analysis of the μm marker length to the measured image of the prototype sample from the micrograph. Depending upon the magnification range, a pitch structure of some dimension was available in the micrograph, for measurement and comparison (Table 2). The second study was a comparison of the measured image to the NIST (FESEM) measurements of the same structure on the same sample.

**Fig. 7:**
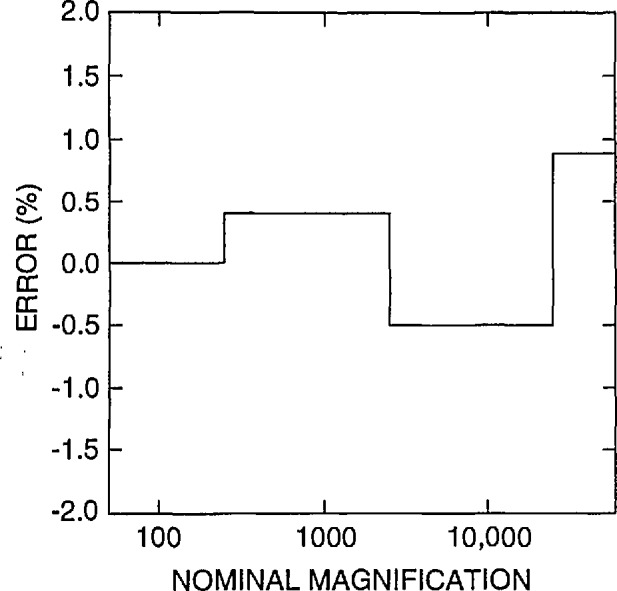
Schematic plot of the decade magnification of an SEM showing a distinct transition between the decade points leading to large jumps in the magnification at the transition points when miscalibrated.

There are three fundamental calibrations that alter either the SEM magnification or the apparent magnification for many “laboratory” scanning electron microscopes. These calibrations, therefore, have direct bearing upon the results of this study. The first and foremost is the adjustment of the X and Y column scans. This adjustment is often done manually with calibration potentiometers at the board level by the field service engineer to some type of standard. In the more modern instruments, some of these adjustments may be under software control but usually there is at least one manual potentiometer adjustment. This adjustment sets the column scans (i.e., magnification); and this adjustment is often, but not always performed in decades, such as: lowest magnification to 250 ×; 260 × to 2500 × and so on throughout the range. The transition between decades must be made as smooth as possible within the adjustability of the potentiometer or software step. Otherwise gross or “sawtooth” jumps in magnification can be seen as the magnification is increased or decreased (Fig. 7). For the decade transition to be smooth, measurement of the pitch of a defined structure at the high end of the lower decade (i.e., 2400 ×) should equal the pitch measurement of the same defined structure at the low end of the next decade (i.e., 2500 ×). The graphical magnification data from the participants shown here in this report would best be represented as decade jumps—if the transition points were known for all instruments. Unfortunately, this information is not known for all the instruments, so the data are plotted with a line connecting the points. Thus, any large jumps in magnification between data points are not emphasized.

The standards used for the calibration of the instruments used by the participants in this study were quite varied. By far the majority (over 50%) used NIST SRM 484 but other “standards” included: latex spheres, in-house standards, and copper transmission electron microscope grids. Of course, some participants used no standards or did not know if their instrument was calibrated to a standard sample.

**Fig. 8:**
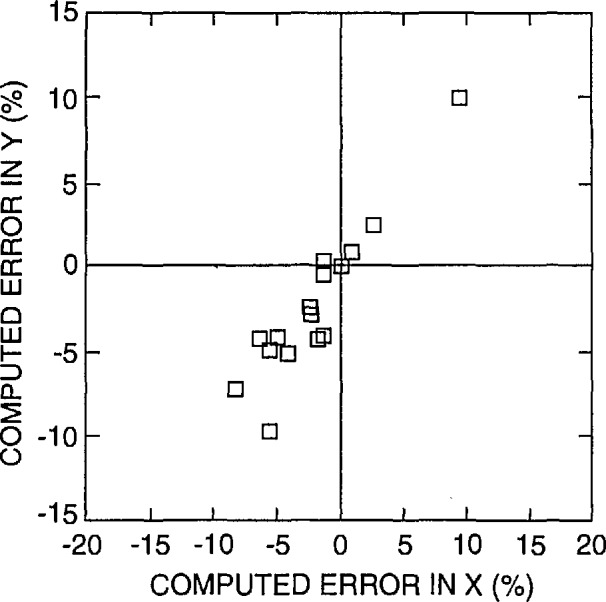
A plot of the measurement of the X and the Y magnification calibration error for high accelerating voltage operation obtained from the low magnification images. Plotted is the computed error (%) from the expected value for X plotted against the computed error (%) from the expected value for Y. Perfect X and Y calibration would place the boxes representing the data points in the center of the graph (0,0).

The ratio of the calibration measurement of the X to the Y scan should be 1:1. Deviation from this relationship makes round structures appear oblong and square structures appear rectangular. In this paper, this characteristic is referred to as the squareness of the image. This definition does not take into account any other factors that could also distort the image such as pincushion distortion or skew. A measurement of the X and the Y magnification calibration was obtained from the lowest magnification images (60 ×) provided by the participants. Figure 8 shows the results of that measurement. Plotted is the measured error (%) from the expected value for both X and Y. Few instruments involved in this study had the X to Y ratio at (or even near) the desired 1:1. A perfect calibration would fall in the center of the graph (0,0). It is apparent that at low magnification, the basic calibration of the squareness of an SEM is inadequate. One reason for this problem is that it is very difficult to match the proper X and Y potentiometer settings due to insensitivity (coarseness) of the adjustment potentiometers. A second problem is that the calibrated lines of NIST 484 are too small to be used to adjust the low magnifications and no large pitch dimension is available. Therefore, a secondary calibration standard such as a transmission electron microscope grid is often used for the low magnification calibration. This is why the approximately 3 mm low magnification pattern was included in the new prototype standard used in this study.

**Fig. 9:**
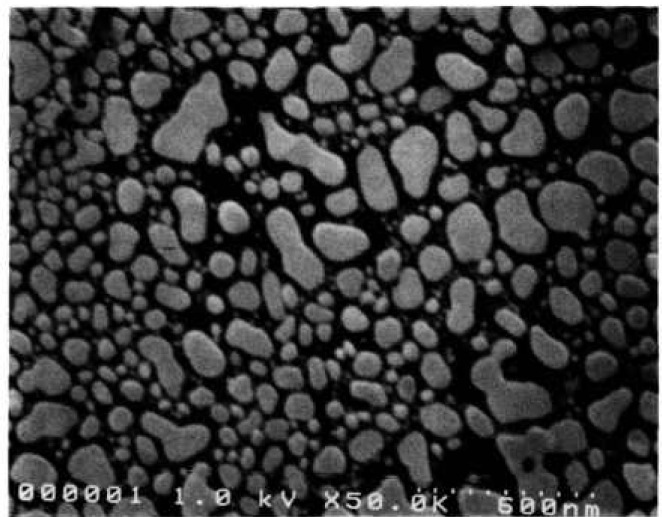
Micrograph showing the calibrated μm marker represented here as series of small white squares. The pitch between the first and the last square represents 600 nm, as discussed in the text. (Reductions during the publication process may change the value.)

The second calibration of interest is the adjustment of the photographic CRT. Since, for many laboratory SEMs, the final record is the micrograph, the calibration of the photographic CRT is critical. The major calibration of the photographic CRT is associated with the adjustment of the alphanumerics especially the micrometer marker. The micrometer marker is generally the measurement fiducial used by the recipient of the micrograph to determine the size of structures in the micrograph. Even if the column scan calibrations are correct, erroneous measurement data can be generated if the micrometer marker is incorrectly calibrated. Figure 9 shows a micrograph where the micrometer marker (represented as a series of small white squares) has been adjusted to be exactly 30 mm in pitch from the left edge of the far left block to the left edge of the far right block. Based upon this, the length of that marker should be equal to 600 nm at a correctly calibrated magnification of 50 000 ×. This adjustment was very accurately done using the Isaac system, but field service engineers do not have the availability of such systems for calibrations on-site in most SEM laboratories.

The third calibration step is the adjustment of the visual CRTs so that the image viewed and focused is reasonably equivalent to the photographed image. This calibration has no bearing upon the column magnification per se but is aesthetically necessary so that the visual image field that the SEM operator sees is equivalent to that which is photographed.

The dedicated “linewidth measurement” instruments or those with linewidth measurement computer systems also have an added calibration in the software of the measurement function. This places a user defined “offset” or “correction” factor into the system. This offset can be determined from measurement of an internal standard, NIST standard or even the pitch of the actual device. Unfortunately, this offset usually does not effect the actual column scans or any of the above mentioned calibrations—only the “computer” measurement made directly with that system. Therefore, digital measurements made with the computer system may be relatively correct, but micrographs taken with that system may be out of calibration by several percent. This software adjustment is really a point calibration in that it is usually done in the decade where the measurement is to be made. Erroneous results can also occur if the magnification is changed from that “calibrated” decade without rechecking the point calibration for that new decade.

### 3.1 Image Magnification/Micrometer Marker

**Fig. 10:**
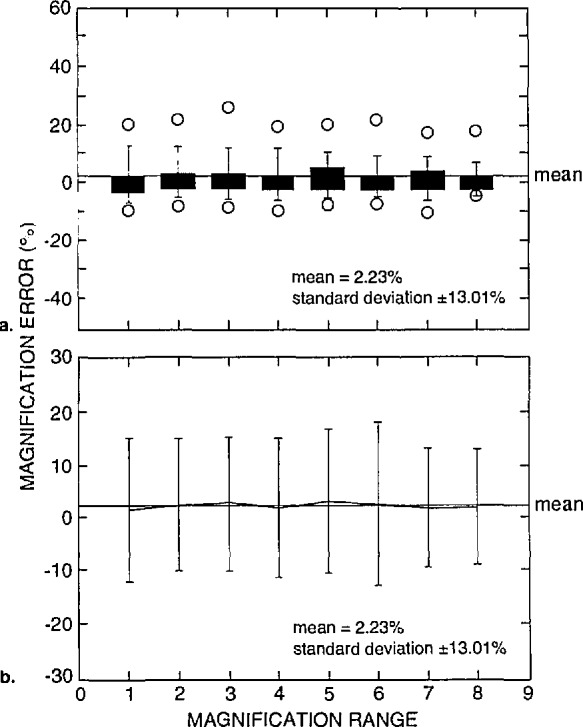
Micrometer bar error, (a) Box plots and (b) scatter plots of the percent error of the measured structure in the micrograph to the length of the μm bar for the eight magnification ranges, all instruments at all accelerating voltages.

**Fig. 11:**
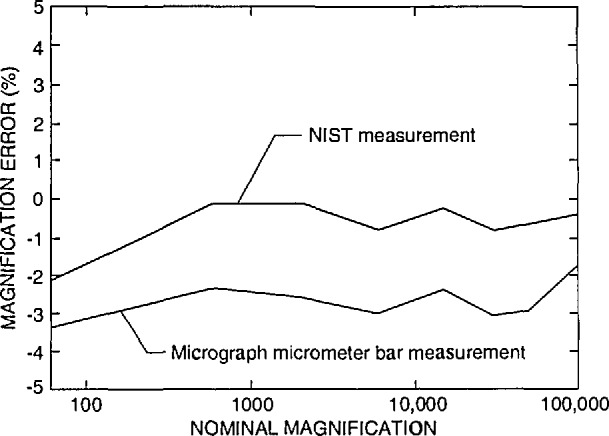
Micrometer bar error. Plot showing the relationship typical of the error of the micrometer bar measurement to the NIST measurement of the same structure for one set of data.

Overall, all the SEMs involved in this study demonstrated some error in the adjustment of the micrometer bar. This is a very difficult adjustment to make since it is made directly from the micrograph, often from a relatively short fiducial line (often 10–30 mm in length). Box plots of the percent error demonstrated by all the instruments of this study relative to the magnification range (for all accelerating voltages reported) are shown in Fig. 10a. The box of the plot shown encompasses the 25th through the 75th percentiles of the data. The lines making up the box plot represent the 10th, 25th through 75th, and the 90th percentiles. Data of either the 5th and 95th percentiles are shown as a symbol (0) above or below the 10% and 90% lines. The mean of the error of these measurements was 2.23% with a standard deviation of ±13.01%. The individual means and standard deviations for each magnification range are shown in Fig. 10b. Where these data are concerned, it could be argued that statistically, the mean may not be the most appropriate description since the distribution is nonsymmetric. But, for this study, the mean has been adopted since it is the most common manner to describe this type of data. It should be understood that the calibration of the micrometer bar is extremely important because even if an SEM is properly calibrated for the column scan magnification, measurement results can be in error if they are obtained from a comparison to a miscalibrated micrometer bar. In general, this represents a slight offset (either positive or negative) to the NIST measurements (discussed below) depending upon how far the micrometer bar calibration is miscalibrated (Fig. 11).

### 3.2 Image Magnification/NIST Measurements

NIST SRM 484 has an uncertainty of about 0.05 μm for the nominal 1 μm pitch or about 5%; therefore, for these comparisons a +5% upper tolerance (UT) and a −5% lower tolerance (LT) was established leading to an overall 10% possible “acceptable” error range. Until recently, SEM manufacturer’s specifications for magnification calibration within 10% were considered to be acceptable because no calibration sample better than this was available. With the new SEM magnification prototype sample, sufficient structure is available to test the entire magnification range of most SEMs with a high degree of accuracy.

**Fig. 12:**
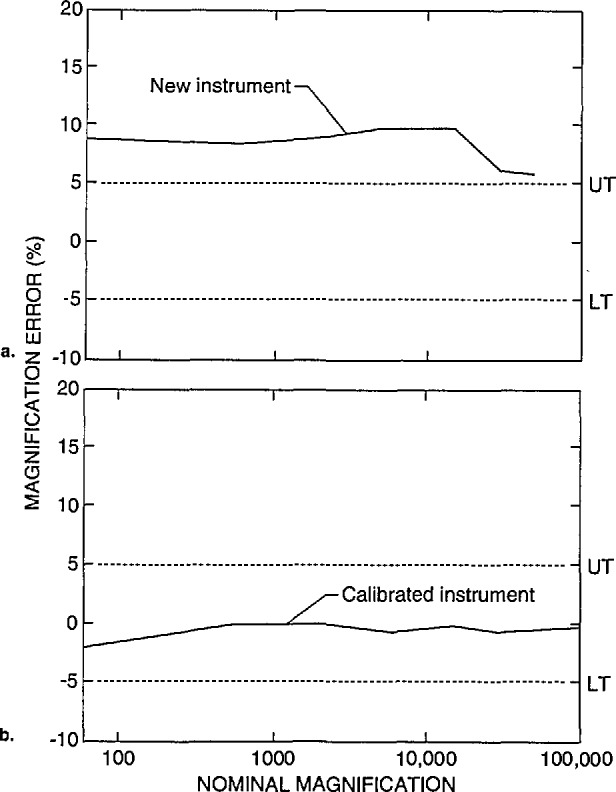
Magnification calibration, (a) Newly delivered instrument demonstrating the uncalibrated nature of the instrument, (b) Well calibrated instrument of the same model from a different laboratory.

Data obtained from a new instrument are shown in Fig. 12a. This instrument was recently installed, and it is unlikely that any magnification checks were run on the instrument. This instrument is demonstrating a systematic offset in magnification of, on average about, *+*9% up to about 30 000 × and slightly less error above 30 000 ×. With calibration, a similar model instrument submitted by another participant is shown to be calibrated within about ±1% or well within the above defined “common” specification (Fig. 12b). Differences of sensitivities between the resistors of the decades and care taken during the adjustment procedure still leave some irregularities in the profile, but, this performance compares favorably with the NIST instrument calibration (Fig. 2).

**Fig. 13:**
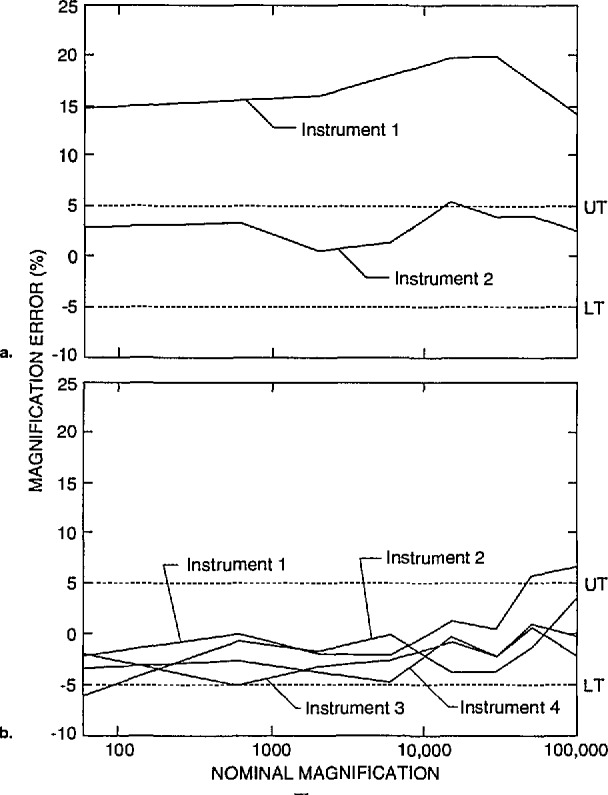
Comparison of the magnification calibration of instruments from the same site, (a) Site where two Instruments are not In agreement with each other, (b) Site where a good deal of agreement exists between instruments.

Comparison of the magnification of instruments from a single site can be seen in Fig. 13. Figure 13a shows the results from two instruments from the same laboratory using the same data conditions. From the graph it can be seen that the two instruments vary nearly 10% in magnification from each other. Another site is shown in Fig. 13b where there is a reasonably tight agreement between the four instruments tested and the entire group of instruments generally fell within the acceptable range. It is apparent from this plot that these four instruments would provide similar results between the range of 1000 × to about 20 000 × magnification.

**Fig. 14:**
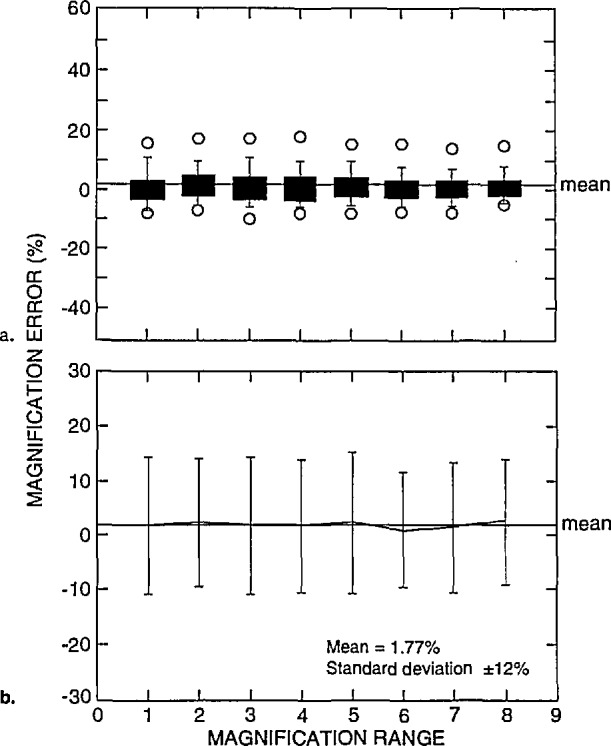
Magnification calibration error, (a) Box plots and (b) scatter plots of the magnification error for all the ranges as compared to the NIST measurements for all instruments and accelerating voltages.

The graphical representation of the magnification error as compared to the NIST measurements (relative to the magnification ranges for all the instruments tested in this study) are shown in Fig. 14. Figure 14a represents box plots of the magnification error data obtained from all the instruments. In this figure, the mean of the error of these measurements was 1.77% with a standard deviation of ±12.03%. The individual mean and standard deviation for each magnification range is shown in Fig. 14b. This figure is directly comparable to the data set of Fig. 10.

**Fig. 15:**
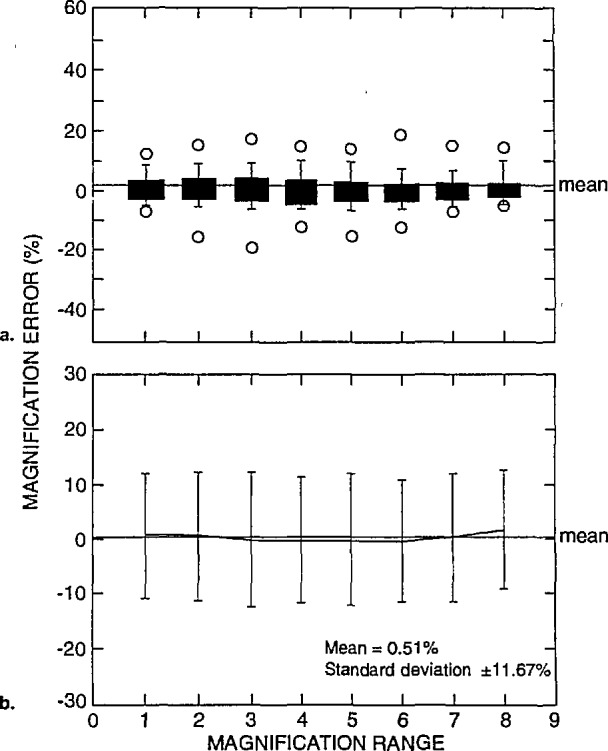
Magnification calibration error as related to accelerating voltage, (a) Box plots and (b) scatter plots of the magnification error for the highest reported accelerating voltages.

**Fig. 16:**
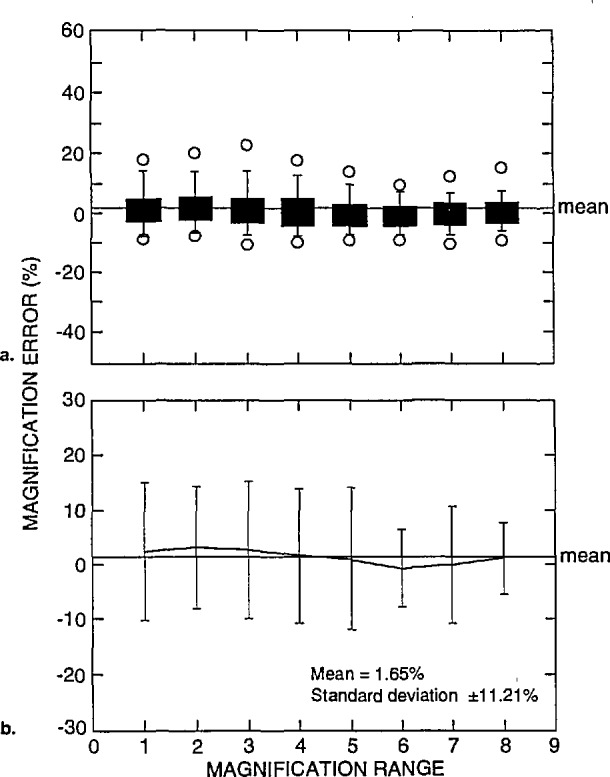
Magnification calibration error as related to accelerating voltage, (a) Box plots and (b) scatter plots of the magnification error for the lowest reported accelerating voltages.

The data described above in Fig. 14 can be separated and compared relative to the instrument’s accelerating voltage performance, as shown in Figs. 15 and 16. Figure 15a represents box plots of the data obtained from the highest accelerating voltage reported from each instrument. In this figure, the mean of the error of these measurements was 0.50% with a standard deviation of ±11.67%. The individual mean and standard deviation for each magnification range at high accelerating voltage is shown in Fig. 15b. In comparison, Fig. 16a represents box plots of the data obtained from the lowest accelerating voltage reported from each instrument. In this figure, the mean of the error of these measurements was 1.65% with a standard deviation of ±11.21%. The individual mean and standard deviation for each magnification range for low accelerating voltage is shown in Fig. 16b. Comparison of these data for high keV operation (Fig. 15) to that for low keV operation (Fig. 16) demonstrates that the error increases overall at the low accelerating voltages. This is expected since NIST SRM 484 is commonly used at high accelerating voltage and no NIST low voltage SEM magnification calibration sample is currently available.

**Fig. 17:**
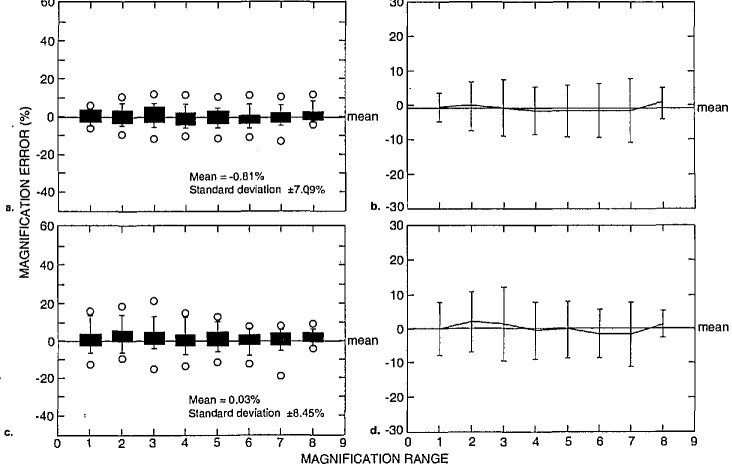
Magnification calibration error as related to type of laboratory and accelerating voltage, (a) Box plots and (b) scatter plots of the magnification error for semiconductor related laboratories for the highest accelerating voltages reported, (c) Box plots and (d) scatter plots of the magnification error for semiconductor related laboratories for the lowest accelerating voltages reported.

**Fig. 18:**
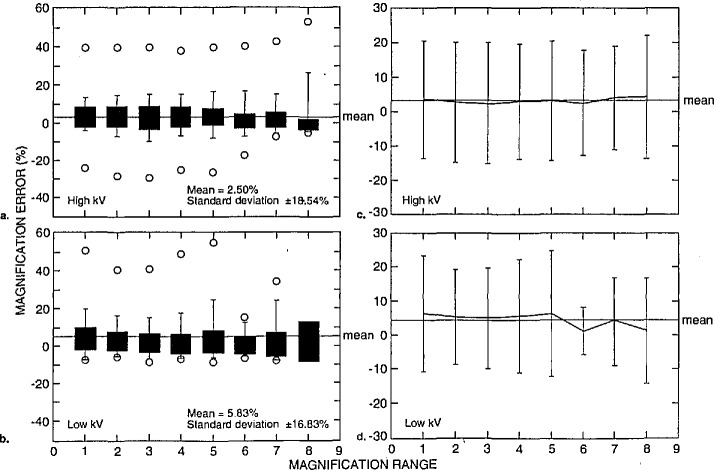
Magnification calibration error as related to type of laboratory and accelerating voltage, (a) Box plots and (b) scatter plots of the magnification error for nonsemiconductor related laboratories for the highest accelerating voltages reported, (c) Box plots and (d) scatter plots of the magnification error for nonsemiconductor related laboratories for the lowest accelerating voltages reported.

These data can be separated even further in order to determine the magnification calibration performance of the semiconductor industry participants to other non-semiconductor related laboratories. Figure 17 represents the data obtained from semiconductor industry participants and Fig. 18 represents data from other non-semiconductor related laboratories. Figure 17a is box plots representing the data from the semiconductor related laboratories of the highest accelerating voltages reported from each instrument. In this figure, the mean of the error of these measurements was −0.81% with a standard deviation of ± 7.09%. The individual mean and standard deviation for each magnification range for the high accelerating voltage performance is shown in Fig. 17b. Figure 17c is box plots representing the lowest accelerating voltage reported from each instrument from these laboratories. In this figure, the mean of the error of these measurements was 0.03% with a standard deviation of ±8.45%. The individual mean and standard deviation for each magnification range for the high accelerating voltage performance is shown in Fig. 17d. These data are contrasted to the performance of the “other” participants. Figure 18a is box plots representing the highest accelerating voltage reported from each instrument from the nonsemiconductor related laboratories. In this figure, the mean of the error of these measurements was 2.50% with a standard deviation of ±18.54%. The individual mean and standard deviation for each magnification range for the high accelerating voltage performance is shown in Fig. 18b. Figure 18c is a box plot representing the lowest accelerating voltage reported from each instrument. In this figure, the mean of the error of these measurements was 5.83% with a standard deviation of ±16.83%. The individual mean and standard deviation for each magnification range for the low accelerating voltage performance is shown in Fig. 18d. It should be noted that the “other” category included the data from the applications laboratories from three SEM manufacturers and, thus the overall error was somewhat reduced. Results from all of the data sets including the maximum error reported is found in Table 3.

### 3.3 Accelerating Voltage Compensation

**Fig. 19:**
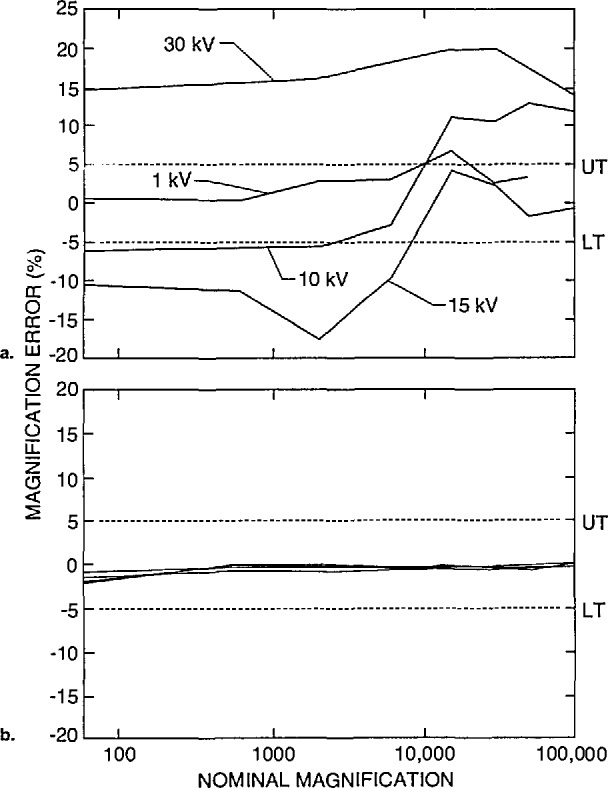
Comparison of the error of the accelerating voltage compensation as related to magnification for two different instruments, (a) Four different accelerating voltages on instrument 1 showing poor compensation for accelerating voltage changes, (b) Four different accelerating voltages on instrument 2 showing excellent accelerating voltage compensation.

**Fig. 20:**
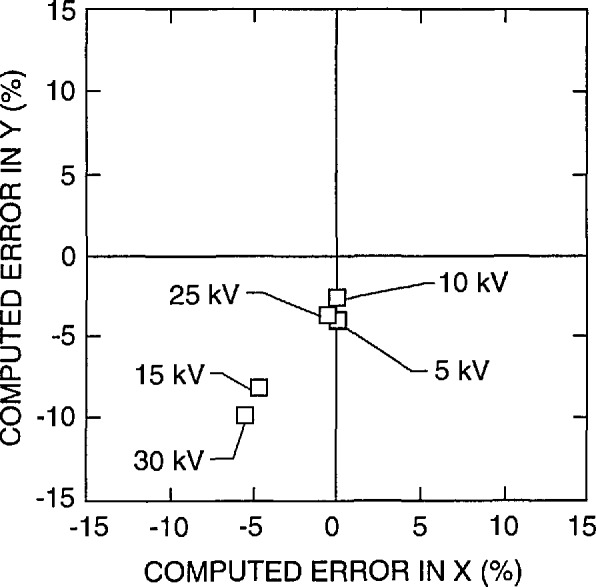
X-Y Compensation error as related to accelerating voltage. A comparison of the error of the X to the Y measurement as related to the expected value is compared for several accelerating voltages for the same instrument. Perfect X and Y calibration would place the boxes representing the data points in the center of the graph (0,0) and perfect compensation correction would overlay each of the boxes at each accelerating voltage.

An analysis of the performance of the instrument accelerating voltage compensation circuitry was also obtained from the supplied data. It is assumed by most operators that when the accelerating voltage is changed, the magnification compensation circuitry adjusts for this change and the magnification is correctly adjusted. Many factors which are outside of this study complicate this process. However, one major factor contributing to variations in the magnification between accelerating voltages is lens hysteresis. Many newer instruments have mechanisms such as degaussing circuitry to compensate or correct for this problem. Figure 19a shows the performance of an older instrument at four separate accelerating voltages. Note that there is at least a 5% error spread between each accelerating voltage range. Figure 19b demonstrates the results from a newer instrument from the same laboratory. Note the tight spread of results. With this instrument, consistent results between accelerating voltages were obtained. The lens compensation effect is also related to the X-Y squareness of the low magnification image as shown in Fig. 20. In this figure, a comparison of the error of the X and Y measurement as related to the expected value is compared for several accelerating voltages for the *same* instrument. As with Fig. 8, perfect X and Y compensation would place the boxes representing the data points in the center of the graph (0,0).

### 3.4 “Linewidth Measurements”

**Fig. 21:**
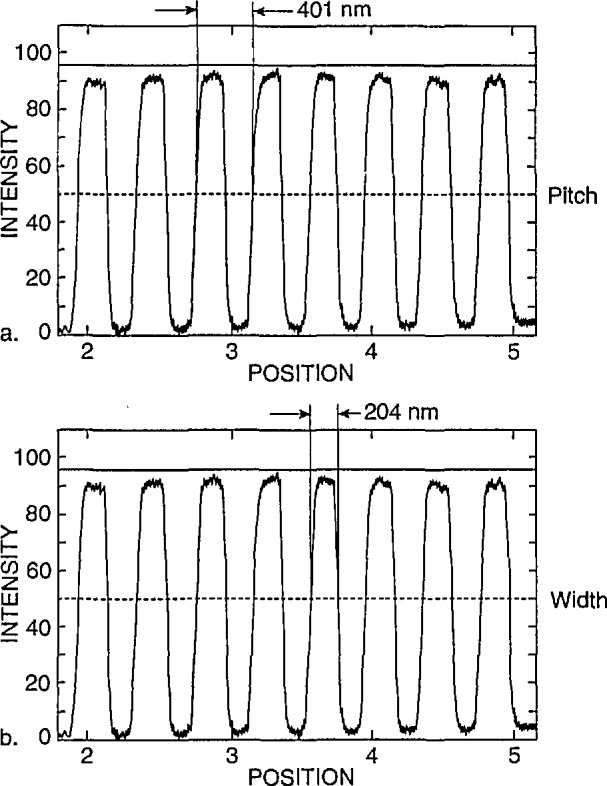
NIST laser interferometer-based metrology instrument measurement scan data, (a) Pitch of 401 nm. (b) Width of 204 nm. Measurements are based on an arbitrary 50% threshold crossing algorithm and have been measured from the collected backscattered electron signal.

**Fig. 22:**
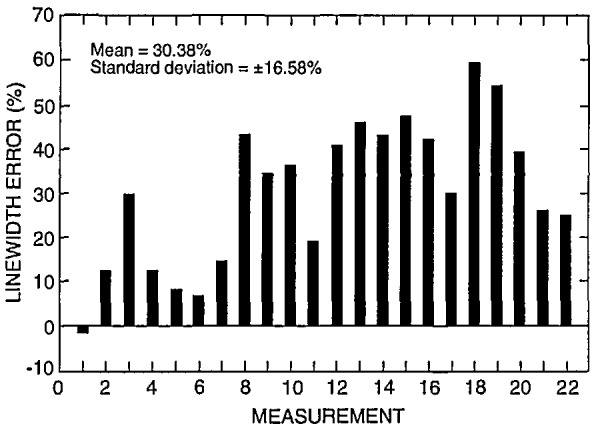
Plot of the error of reported “linewidth” to that measured by the NIST metrology instrument for the 22 separate measurements reported.

**Fig. 23:**
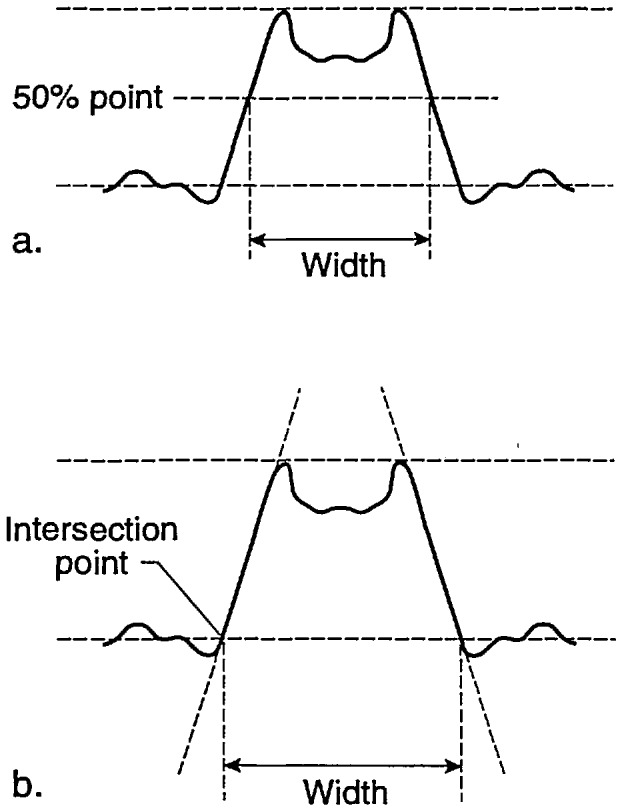
Diagrammatic Comparison of the difference between two common measurement algorithms on the reported width measurement (a) Threshold crossing algorithm, (b) Linear approximation algorithm.

The NIST prototype SEM sample is designed to be used for calibration of the SEM magnification to a known pitch. This sample is not designed nor is meant to be used as a “linewidth” calibration sample. The reasons for this distinction have been discussed extensively in the literature. However, one exercise requested of the participants was to report their “best-guess” of the width of the 0.2 (μm nominal lines. Comparison measurements from one of the NIST samples were performed on the NIST metrology instrument at high accelerating voltage (the current configuration of the instrument) using the laser interferometer stage. The laser interferometer measurement of one of the samples reported an average pitch of 401 nm and an average linewidth of 204 nm. Multiple lines were used to obtain the average since it was unknown which lines were measured by the participants. Using the NIST metrology SEM, plots of the video to the laser data representing 24 000 data points for the backscattered electron image are shown in Fig. 21. Measurements were obtained using an arbitrary 50% threshold crossing algorithm. These measurements compare within 3 nm of another set of data submitted by one of the participants using a similar laser interferometer based metrology instrument. The average measurement of these lines was used as the “standard nominal” measurement and the data supplied by the participants was compared to that number and the error plotted (Fig. 22). In some instances, measurements of the same lines using the same fundamental instrument conditions but a variation in accelerating voltage by the participants metrology instruments demonstrated differences of as large as 31 nm (315 nm at 1 keV and 284 nm at 2 keV). This variation in measurement results, especially between different accelerating voltage is expected and has been demonstrated on other types of samples [11]. Other possibilities for variation include: electron beam interaction effects, differences between secondary and backscattered electron measurements, electron beam diameter differences between instruments, the effect of sample contamination, the differences between measurement algorithms and sample variability. For example two common algorithms used for the determination of the data for this work were the threshold crossing algorithm and the linear approximation algorithm. Figure 23 shows a comparison between measurements made between the two methods. Clearly, a “standard” measurement algorithm should be developed. This algorithm should be designed so it can be used on any SEM linewidth measurement instrument. Using this algorithm, the measurement data would be handled in an identical manner irregardless of the instrument for comparison purposes. The differences reported for “linewidth” underscores the fact that the magnification cannot be “point calibrated” to a linewidth type sample, and a magnification type sample cannot be used as a “linewidth” calibration sample unless electron beam interaction modeling is capable of predicting the accurate location of the edges, within some uncertainty, for various instrument and sample conditions.

### 3.5 Specimen Contamination

Sample contamination is inevitable. Contamination results from sample handling, the environment and the instrument. Hydrocarbons interact with the electron beam and form a layer on the surface. The speed at which this deposition occurs varies with the amount of hydrocarbon (or other contaminant) available to interact, as well as, the operational conditions of the instrument.

**Fig. 24:**
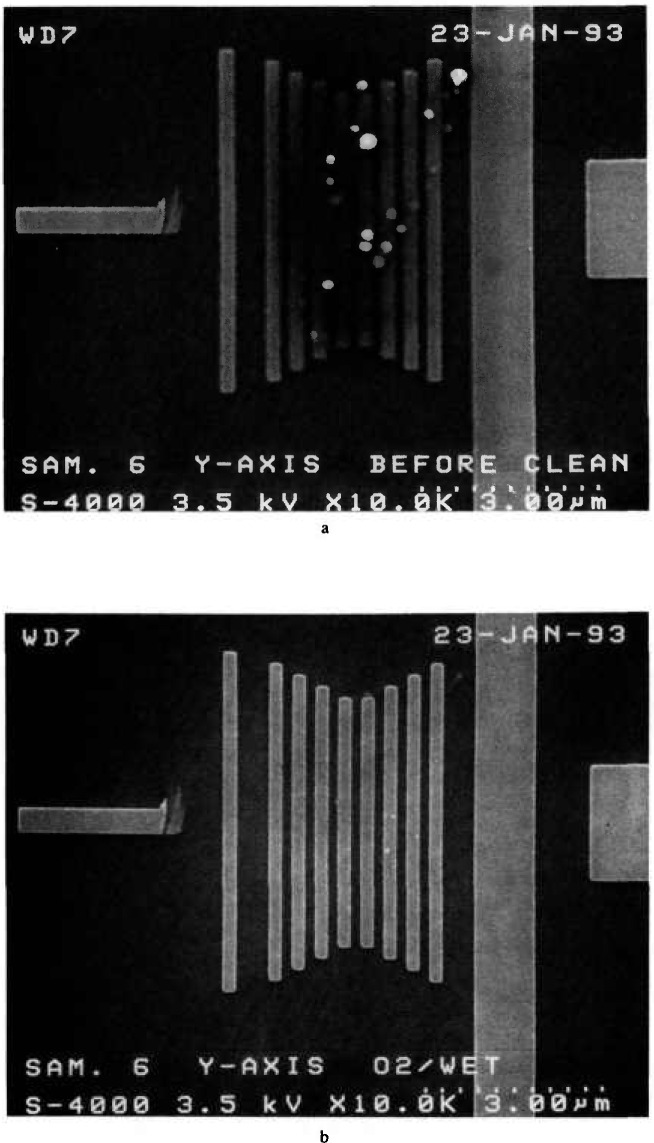
Contamination micrographs, (a) Micrograph demonstrating the condition of a sample as received from one participant of the study, (b) Micrograph of the same sample after cleaning. (Micrographs courtesy of Al Pelillo, Digital Equipment Corporation).

In this study, few fully dry-pumped SEMs were used to examine the samples. Dry pumped in this instance is defined as a system equipped with a magnetically levitated turbomolecular pump which is backed by a molecular drag type pump as well as a molecular drag-type roughing pump on the sample exchange chamber. In one controlled instance, a sample from the first round, (therefore un-examined), was directly inspected in the dry pumped system with no resulting contamination deposition on the surface. The same sample was placed into another “clean” but, non-dry pumped system and rapid sample contamination resulted. From this experience it became apparent that a cleaning procedure was needed. With the assistance of Mr. Aldo Pelillo of Digital Equipment Corporation a successful cleaning procedure was developed. The samples were cleaned in oxygen plasma in intervals using power output ranging from 100–250 W, depending upon the contamination level. It was demonstrated that most of the contamination is removed within the first two cleaning cycles. With the higher wattage, some of the samples tested tended to oxidize requiring a follow-up wet cleaning of the surface in dilute hydrofluoric acid (10:1 – DiH_2_O:HF for 1.5 min). This procedure was applied to samples measured in their laboratory with great success. Samples measured and returned by some of the participants were inspected at low accelerating voltage at NIST and then sent to be cleaned. The resulting micrographs are shown in Fig. 24.

## 4. Conclusion

The results of the NIST interlaboratory SEM study underscores that each SEM must be considered as an individual unit. Calibration and adjustment is necessary and must be checked and re-checked periodically in order to make sure that the data obtained from the instrument are correct.

Throughout this study, it became apparent that the magnification calibration capability for the current, more modern instrumentation is far better than for the older instruments. However, the sensitivity of this adjustment should be far finer. Calibration potentiometers which are quite often “5-turn” variable resistors, do not have sufficient sensitivity to properly adjust the transition points adequately for the precision needed for modern SEM operation, especially those used for metrology. Changing these variable resistors to 10 or 20 turn potentiometers would be a step in the right direction, but this is only is part of the story. The entire calibration/scan system of the SEM should be redesigned for improved precision for both magnification calibration and accelerating voltage compensation. The 10% rule no longer applies and we should strive for the 0.5% or better rule.

The applicability of the SEM prototype sample has been proven through this study. The prototype sample, as previously described and published, or a sample identical to the test samples used in this study could be issued as an SRM. However, several excellent suggestions made by the participants during the course of the study will be incorporated in the final standard. The first suggestion is that there be more calibration patterns available since contamination (even with the availability of the cleaning procedure) is Inevitable. A newly designed pattern including four fine calibration patterns, two in X and two in Y has been designed. It is planned that NIST will certify one pattern in X and one pattern in Y. It will then be up to the user to secondarily calibrate and use the other patterns. The lines have also been lengthened somewhat. Another improvement is that an array of the focusing and astigmatism correction marks has been included near to the fine patterns.

NIST does not, at the current time, have a semiconductor processing facility capable of manufacturing the new proposed SEM magnification sample. NIST does however, have the measurement capability to measure and certify the new standard. Therefore, NIST must rely on commercial state-of-the-art semiconductor processing facilities to fabricate the samples. Until recently only a small number of these facilities were capable of making the standard and a smaller number of those were willing to undertake the challenge. A similar situation occurred with the manufacture of the Optical Photomask Standards SRM 473, 474, and 475. All of these standards push the state-of-the-art of device fabrication to the limit. Specifications for wall verticality and edge roughness are extremely tight and place demands on the fabrication facility that are not required by normal chip production. For the SEM magnification sample, the NNF of Cornell University has been extremely cooperative in assisting in the fabrication of the samples for this and the previous study—but they are not a production facility. The task of the NNF was to prove the sample could be made and they succeeded in that task, but it was not their task to produce it in production quantities. NIST/Cornell demonstrated the concept of this magnification standard in 1988, but it has taken until just recently to identify commercial companies interested and capable of making the standard. Currently there are at least three companies interested in fabricating the standard and procurement is currently underway.

Sample contamination is inevitable and a cleaning procedure has been developed with the co-operation of Digital Equipment Corporation. Contamination results from sample handling, packaging, the environment, and the instrument. Hydrocarbons from whatever source interact with the electron beam and form a layer on the surface. The speed at which this deposition occurs varies with the amount of hydrocarbon (or other contaminant) available to interact, as well as the operational conditions of the instrument. Many of the participants of the study commented about the contamination rate of the prototype sample. Some participants were able to cycle the sample successfully through as many as six instruments whereas others stated that the “sample contaminated instantly.” Participants of the first round received virgin samples directly from the wafer fabrication facility. Yet, in all but the fully dry pumped scanning electron microscopes, sample contamination proved to be an issue. Was the contamination being deposited on the sample calibration structures from the packaging, handling or instrument? This is unknown, but, it seems to be an area which should be studied further by all interested parties. Participants of the second round were, unfortunately, working under a hardship since the sample each received was viewed by another participant, and the sample was also checked by NIST before being sent out the second time. If more test samples had been available, this recycling of samples would not have been necessary.

## Figures and Tables

**Table 1:** List of instruments

AMRAY	HITACHI	JEOL
1000B	S-800 (3)	JSM-35 CF
1610	S-4000 (2)	T-330 A
1645 (2)	S-4100 (1)	JSM 848 A(2)
1850 FE	S-7000 (3)	JSM IC 845 (3)
1860 FE	S-6820	JSM 840 FE(2)
1880 FE	S-6100	JSM 6400 FE (2)
BIORAD	S-6000 (5)	JSM 6400
	S-6600	JSM 5400
800 FE	S-900	
Cambridge	TOPCON (ISI)	NANOMETRICS
S-200 (3)	CC-CD	Cwikscan III
S-250	DS-130 FE	PHILIPS
	DS-130	
ETEC		XL-30
OMNISCAN		

**Table 2:** Magnification ranges

Magnification range	Nominal magnification	Measured pitch dimension (μm)
1	60 ×	500
2	600 ×	50
3	2000 ×	25
4	6000 ×	10
5	15 000 ×	5
6	30 000 ×	2.5
7	50 000 ×	1.2
8	100 000 ×	0.8

**Table 3:** Error Summary

Type of error measurement	Mean	Standard deviation	Maximum error
Micrometer bar	2.23%	± 13.01%	−43.42%
Magnification	1.77%	±12.03%	63.08%
All high kV	0.50%	±11.67%	57.71%
All low kV	1.65%	±11.21%	63.08%
Semiconductor high kV	−0.81%	± 7.09%	18.12%
Other high kV	2.50%	±18.54%	57.71%
Semiconductor low kV	0.03%	± 8.45%	33.70%
Other low kV	5.83%	±16.83%	63.08%
Commercial high kV	5.02%	± 4.04%	9.76%
Commercial low kV	2.64%	± 5.20%	10.90%

## Footnotes

1 Certain commercial equipment, instruments, or materials are identified in this paper to specify adequately the experimental procedure. Such identification does not imply recommendation or endorsement by the National Institute of Standards and Technology, nor does it imply that the materials or equipment identified are necessarily the best available for the purpose.
